# Re-enforcing High-Risk Acute Pericarditis Requiring Hospital Admission: An Unusual Case of Critical Idiopathic Acute Pericarditis Presenting As Tamponade and Pleuro-Pericardial Complications in a Patient Presenting With Flu-Like Symptoms

**DOI:** 10.7759/cureus.58147

**Published:** 2024-04-12

**Authors:** Ikpechukwu J Okorie, Muhammed Atere, Annmarie Fernando, Derek Ugwendum, Jay Nfonoyim, Jay Nfonoyim

**Affiliations:** 1 Internal Medicine, Richmond University Medical Center, New York, USA; 2 Cardiology, Richmond University Medical Center, New York, USA; 3 Pulmonary and Critical Care, Richmond University Medical Center, New York, USA

**Keywords:** pleural interventions, obstructive shock, myo-pericarditis, pericardial tamponde, idiopathic pericarditis

## Abstract

Pericarditis is an inflammatory process that affects the pericardium, the fibrous sac surrounding the heart. Acute pericarditis accounts for approximately 0.1% of inpatient admissions and 5% of non-ischemic chest pain visits to the emergency departments (EDs). Most patients who present with acute pericarditis have a benign course and good prognosis. However, a rare percent of the patients develop complicated pericarditis. Examples of complications include pericardiac effusion, cardiac tamponade, constrictive pericarditis, effusive and constrictive pericarditis and, even more rarely, large pleural effusion The occurrence of complicated pericarditis can lead to high morbidity and mortality if not urgently managed in most patients. Our case presents a 60-year-old male that presented to the emergency room with flu-like symptoms. However, the viral panel test was negative. He initially got discharged with supportive care but was brought back to the ED by his wife in a critical, life-threatening state due to pericarditis symptoms complicated by tamponade and shock. His condition required urgent intervention and critical level of care. The patient's course was also complicated by myopericarditis and recurrent bilateral pleural effusions, which required therapeutic interventions. This unique case presents the patient group that develop multiple life-threatening complications of acute pericarditis, including cardiac tamponade and shock, affecting several end organs. This case also highlights clues to the predisposing factors to complications of acute pericarditis. Patients who present with high-risk signs and symptoms indicating poorer prognosis warrant further observation and admission. This will also add to the literature reviews regarding the risk factors associated with development of complicated acute pericarditis. This will also serve as a review of pathophysiology, etiology, current diagnosis and available novel treatment for such patients.

## Introduction

Acute pericarditis is the rapid inflammation of pericardium and is seen in up to 5% patients who present to the emergency department (ED) with chest pain [1,2]. Pericarditis is the most common form of pericardial disease and a relative common cause of chest pain [1]. The pericardium has two layers, the visceral and fibrous layer. The pericardial sac normally contains small amount of fluid (<25 to 50 ml) which increases the efficiency of the heart [2]. Pericarditis can be divided into acute, subacute, chronic and recurrent. Most acute pericarditis are self-limiting, although a small percentage can progress rapidly to further complications of pericardial syndrome. Inflammation of the pericardium can result in the accumulation of fluid inside the pericardial sac and develop pericardial effusion. Large pericardial fluid collection or rapid accumulations may compress the chambers of the heart leading to hemodynamic instability and development of shock requiring emergent intervention [2]. Acute pericarditis has also been complicated with pleural effusion, the effusion commonly left sided and is usually a small effusion. Other rare complications include constrictive pericarditis and effusive-constrictive pericarditis.

This case presents one of the rare presentations of idiopathic acute pericarditis with multiple critical complications. Our patient presented with shortness of breath, chest pain and lethargy on the second visit. These symptoms, when combined with echocardiographic finding of pericardial fluid and elevated inflammatory markers, are associated with poor prognosis. Definite diagnosis of acute pericarditis requires a thorough physical exam, electrocardiogram (ECG), echocardiography showing pericardial effusion, and chest x-ray. Although challenging, acute pericarditis has to be differentiated from ST-elevated myocardial infarction (STEMI) because of similar ECG characteristics. Certain presentation features, such as high fever, subacute course and poor response to non-steroidal anti-inflammatory drugs (NSAIDS), may indicate poorer prognosis and will require a longer hospital course. Treatment depends on the etiology. In idiopathic or virally induced type, treatment commonly involves use of NSAIDS. Colchicine can be used as adjunct to control initial episodes and has been associated with about 50% lower recurrence rate [1]. Use of steroids are the second line of treatment, especially for patients not responding, intolerant or contraindicated to NSAIDs and colchicine. There is a 30% chance of recurrence in patients who do not receive preventive therapy [2].

## Case presentation

**Figure 1:**
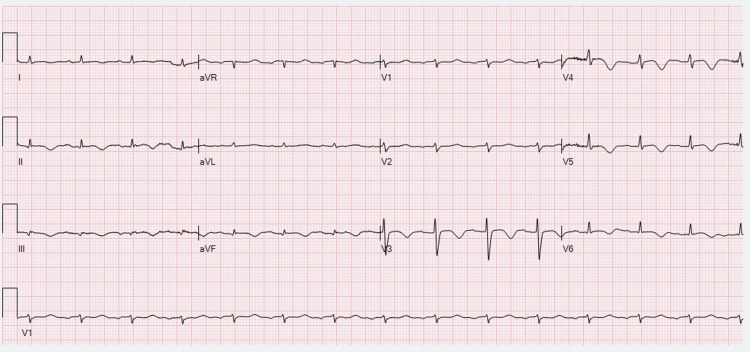
Day 1 ECG in ED showing sinus tachycardia with diffuse ST segment elevation and low voltage QRS ED: Emergency department

**Figure 2:**
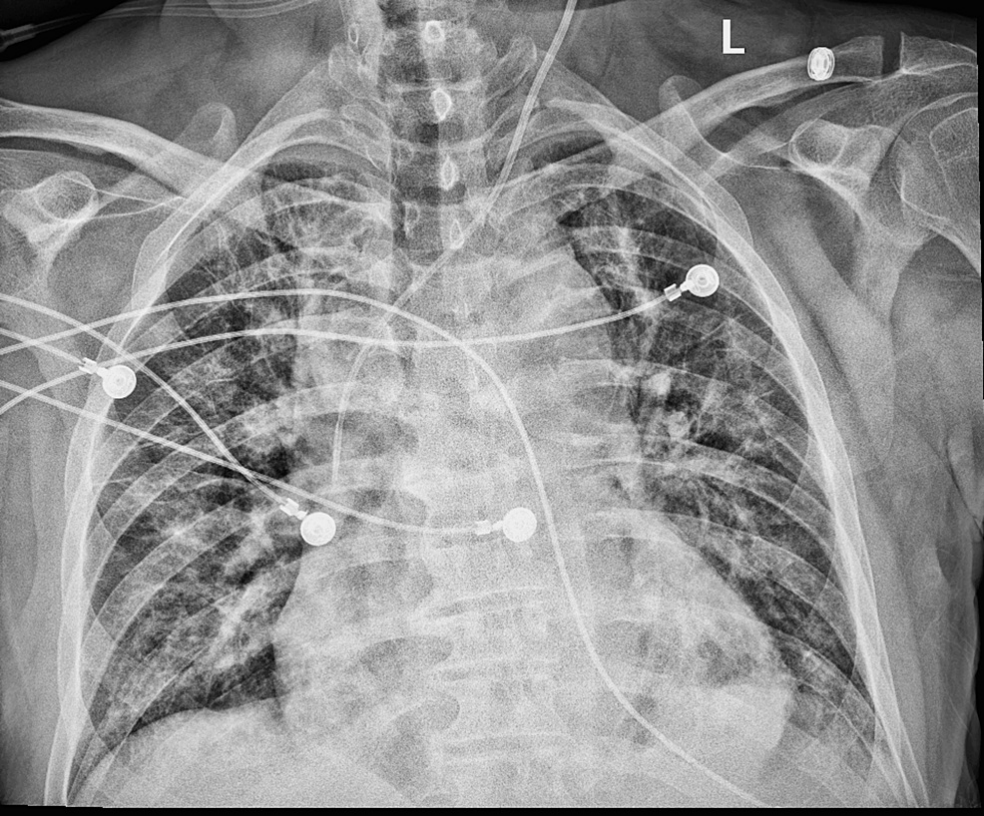
Chest x-ray performed at ED showing cardiomegaly and bilateral lung infiltrate ED: Emergency department

**Figure 3:**
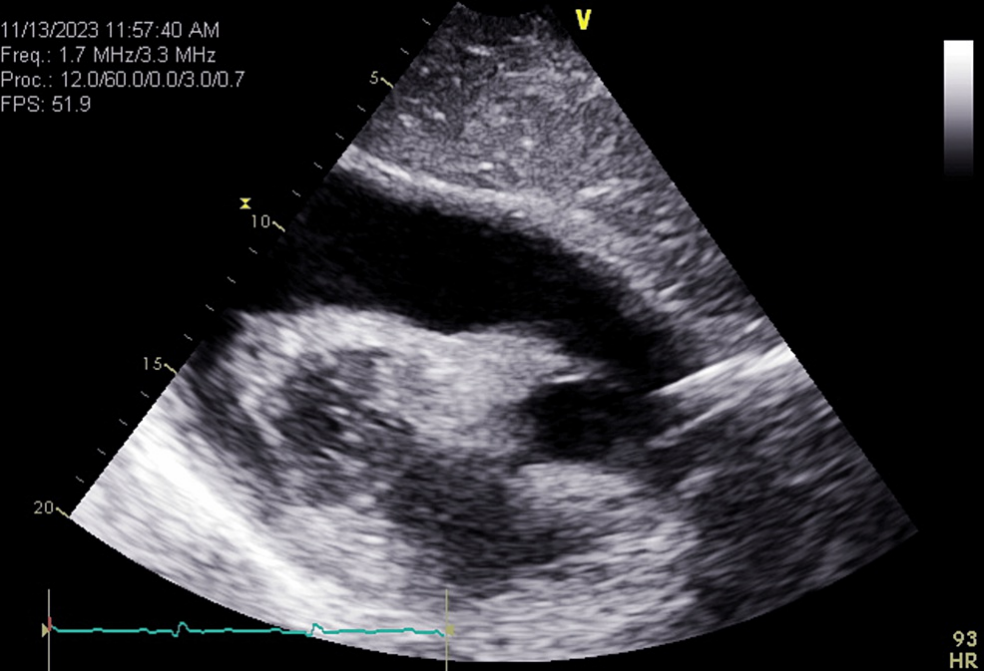
Bedside echocardiogram showing large pericardial effusion in the ED, Day 1 ED: Emergency department

A 60-year-old male with a history of renal cell carcinoma (RCC) post resection three years ago and hypothyroidism was brought in by his wife to the ED for evaluation of chest pressure, weakness, fever, nausea, vomiting, altered mental status and lethargy. History was obtained from the wife as the patient was weak and too altered to speak. The wife stated that the patient was in the ED a day prior for complaints of sore throat, mild tonsillar redness, fever, generalized weakness and chills for three days. In the previous visit, blood work, cultures and viral panels had been negative for active infection, and he had been symptomatically managed, discharged and asked to follow up with primary care. The wife stated that throughout the night, he had experienced fever and chills, and had began vomiting. The wife had called emergency medical service (EMS) in the morning and had been advised to give antibiotics prescribed for possible *Streptococcus pharyngitis*. She stated that the husband's condition seemed to get worse, which prompted the ED visit. On arrival the patient was altered, lethargic, hypotensive (blood pressure 60/40s mmHg) and tachycardic with heart rate in 120s. When asked about symptoms, he pointed to his chest. Electrocardiogram (ECG) showed diffuse ST-elevation in all leads and tachycardia as shown in Figure 1. Initial chest x-ray performed in the ED showed cardiomegaly as shown in Figure 2. STEMI code was called. Bedside echocardiography by the cardiology team showed large pericardial effusion as shown in Figure 3. The patient was urgently taken to the cardiac catheterization laboratory for pericardiocentesis. He arrived at the cardiac catheterization laboratory with a low blood pressure in the low 50s/30s mm Hg. He was infused with 2 L bolus of crystalloid solution. After appropriate sterilization and draping, ultrasound guided pericardial fluid drainage was carried out. Approximately 500 ml of yellow pericardial fluid was drained out, and the catheter tube was left in place for continuous drain. Sample was sent to the lab for analysis. He received additional 2 L bolus of normal saline for hemodynamic stabilization and was continued on maintenance fluid. As Blood pressure did not improve on fluid resuscitation, levophed 25 mcg was started, and he was placed on cardiac monitoring.

**Figure 4:**
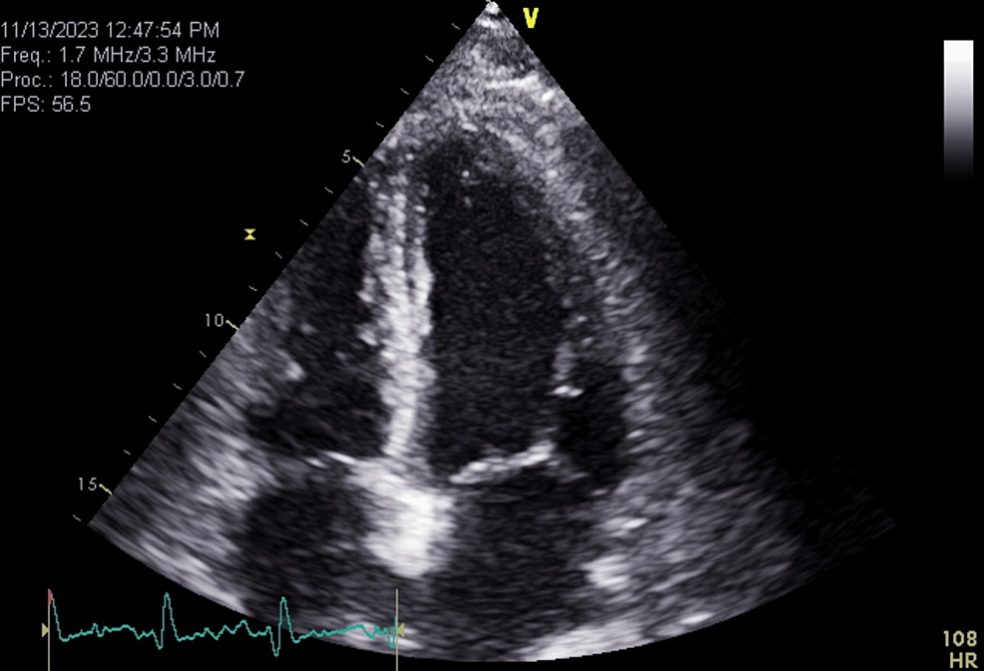
Official echocardiogram after pericardial fluid drain shows mild stable pericardial fluid in cardiac lab, Day 1 post pericardiocentesis

Figure 4 below is the official post pericardiocentesis echocardiogram showing sinus tachycardia , normal left ventricular (LV) size that appears normal to mild reduced LV systolic function. LV ejection fraction appears to be 40% to 45%. The right ventricular free wall appears hypokinetic, and the right ventricular apex was hypokinetic. There appears to be borderline right ventricular systolic dysfunction. There was a medium sized echocardiographic-free space, and no echo evidence of cardiac tamponade. 

Pericardial fluid was sent to the lab for chemistry; cell count and differentials were as shown is Table 1. Further tests carried out on the pericardial fluid include; gram stain, bacteria culture, fungal culture, polymerase chain reaction for Epstein-Bar virus (EBV), cytomegalovirus (CMV), enterovirus and coxsackievirus. The fluid analysis results came back negative. Fluid analysis by Light criteria was indicated of exudative effusion. An infectious disease specialist was consulted. 

**Table 1:** Showing fluid analysis of pericardial fluid on Day 1 of admission, left pleural fluid during Week 2 of admission and right pleural fluid during Week 3 of admission LDH: Lactate dehydrogenase, PMN: Polymorphonuclear neutrophils, WBC: White blood cell count, RBC: Red blood cell count *The reference ranges have not been established for this type of fluid for the test

Fluid Type	Pericardiac Fluid	Left Pleural Fluid	Right Pleural Fluid	Reference	Units
Fluid appearance	Xanthochromatic	Xanthochromatic	Yellow	Clear	-
Fluid pH	7.3	7.7	7.8	*	-
Fluid WBC	5,973	2,073	3,107	*	cells/ul
Fluid RBC	1,000	2,000	1,000	*	cells/ul
Fluid PMN cells	95	79	55	*	%
Fluid mononuclear	3	5	8	*	%
Fluid lymphocytes	12	15	30	*	%
Fluid unclassified cell	-	1	7	*	%
Fluid sodium	-	-	137	*	mmol/L
Fluid potassium	-	-	3.6	*	mmol/L
Fluid glucose	<4	84	63	*	mg/dl
Fluid total protein	4.9	3.3	3.6	*	g/dl
Fluid albumin	3.1	-	2.0	*	g/dl
Fluid LDH	458	122	344	*	U/L
Fluid cholesterol	-	36	-	*	mg/dl
Fluid triglyceride	-	51	-	*	mg/dl

**Figure 5:**
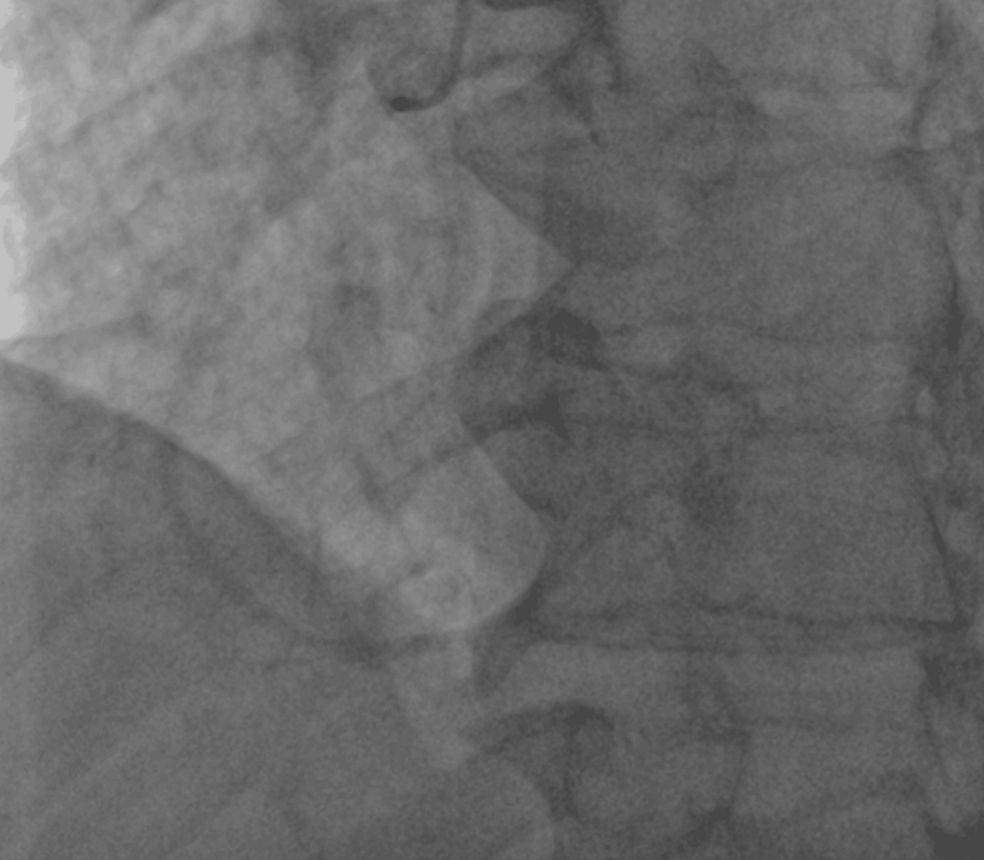
Cardiac catheterization showing diffuse non-obstructive coronary artery disease

Laboratory blood work in ED showed elevated high sensitive troponin 1, as shown in Table 2. Due to chest pain on presentation and echocardiographic finding of mild systolic dysfunction, he underwent coronary angiography to exclude STEMI. Report of the cardiac catheterization was significant for diffuse non-obstructive coronary artery disease of proximal left anterior descending, diagonal, left circumflex, OM1, right coronary artery and 90% focal critical disease of the proximal descending artery as shown in Figure 5.

**Table 2:** Elevated high-sensitivity troponin 1 trend during admission concerning for myocarditis Trop 1 hs: High-sensitivity troponin 1, ED: Emergency department; CCU: Coronary care unit

Test/Timeline	Result	Units	Reference	Comment
Trop 1 hs, day 1 in ED	4,767.2	ng/L	0-54	-
Trop 1 hs trend, day 1, 3H later	52,566.4	ng/L	0-54	Troponin level following cardiac cath.
Trop 1 hs trend, day 2 in CCU	23,339	ng/L	0-54	-
Trop 1 hs trend, day 2, 3H later	15,061	ng/L	0-54	-
Trop 1 hs trend, day 2, 6H later	12, 662	ng/L	0-54	-

The patient was transferred to the coronary care unit (CCU) with the drain in place and output closely monitored. Further lab work, as seen in Tables 3, 4, showed elevated C-reactive protein, lactic acid, high procalcitonin, abnormal liver function test, low hemoglobin level, normal initial erythrocyte sedimentation rate (ESR), low potassium and acute kidney injury. Potassium was corrected. Maintenance fluid and levophed was titrated and continued to maintain mean arterial pressure (MAP) greater than 65 mmHg. He was started on aspirin 650 mg orally every eight hours, colchicine 0.6 mg tablet every 12 hours and proton pump inhibitor for gastric ulcer prophylaxis. He continued nasal cannula oxygen supplementation to maintain stable oxygen saturation. Arterial blood gas showed metabolic acidosis.

**Table 3:** Complete blood count result obtained on Day 1 in the ED WBC: White blood cell count, RBC: Red blood cell count, HGB: Hemoglobin, HCT: Hematocrit, MCV: Mean corpuscular volume, MCH: Mean corpuscular hemoglobin, MCHC: Mean corpuscular hemoglobin concentration, RDW: Red cell distribution weight, ESR: Erythrocyte sedimentation rate, ED: Emergency department

	Result	Reference	Unit
WBC	13.9	4.0-11.2	k/ul
RBC	3.91	4.60-6.10	m/ul
HGB	10.8	13.7-17.5	g/dl
HCT	32.1	40.0-51.0	%
MCV	82.1	79-98	fl
MCH	27.6	25-32	pg
MCHC	33.6	30.7-35.5	g/dl
RDW	13.6	9.9-15.9	%
Platelet count	320	150-400	k/ul
Immature granulocyte	0.4	0-1	%
Neutrophils	58.5	30-71	%
Lymphocytes	28.1	23-54	%
Monocyte	12.0	1-15	%
Eosinophils	2.8	1.6	%
Basophils	0.7	0-1	%
ESR	80	0-15	-

**Table 4:** Blood chemistry test result obtained in the ED during day one of hospital admission AST: Aspartate aminotransferase, ALT: Alanine transaminase, LDH: Lactate dehydrogenase, BNP: B-type natriuretic peptide, TSH: Thyroid-stimulating hormone, TIBC: Total iron binding capacity, Trop 1 hs: High-sensitivity troponin 1

Test	Result	Reference	Units
Sodium	136	136-145	mmol/L
Potassium	3.4	3.5-5.5	mmol/L
Chloride	103	98-107	mmol/L
Carbon dioxide	20	20-31	mmol/L
Anion gap	17	10-20	mmol/L
Blood urea nitrogen	29	7-18	mg/dl
Creatinine	2.74	0.70-1.30	mg/dl
Estimated glomerular filtration rate	24	>60	ml/min
Glucose	44	74-106	mg/dl
Lactic acid	4.6	0.50-2.20	mmol/L
Calcium	8.0	8.3-10.6	mg/dl
Phosphorus	3.2	2.4-5.1	mg/dl
Magnesium	1.73	1.60-2.60	mg/dl
Iron	11	65-175	ug/dL
TIBC	148	250-450	mcg/L
% saturation	7.4	20-50	%
Transferrin	93	188-341	mg/dl
Total bilirubin	2.6	0.2-1.0	mg/dl
Direct bilirubin	0.5	0.0-0.3	mg/dl
AST	144	<34	U/L
ALT	86	10-49	U/L
Alkaline phosphate	96	46-116	U/L
LDH	422	120-240	U/L
Trop 1 hs	4767.2	0-54	ng/L
C-reactive protein	17.1	<1.0	mg/dL
BNP	396	<100	PG/ML
Total protein	6.5	5.7-8.2	g/dl
Albumin	3.5	3.2-4.8	gdl
Procalcitonin	23.73	00.0.05	ng/ml
TSH	3.71	0.55-4.78	Ulu/mL

Due to high suspicion for infection as indicated by leukocytosis, high procalcitonin, elevated lactic acid, fever, hypotension and altered mental status, two sets of blood culture was drawn and a broad spectrum antibiotics of vancomycin and piperacillin-tazobactam were started. Infectious disease doctor added doxycycline to the regimen to cover for common bacterial causes. Blood culture result came back negative. Microbiological investigations including Lyme serology enzyme immunoassay with reflex immunoblot, and Coxiella burnetii titers were negative. Pericardial fluid count showed elevated polymorphonuclear cells, low glucose and elevated lactate dehydrogenase (LDH). The fluid analysis was compatible with exudative acute pericardial fluid. Given low glucose level in fluid analysis, tuberculosis (TB) quantiferon test was sent. Quantiferon was unsurprisingly positive as the patient is from an endemic area, although acid fast bacilli smear and sputum culture were negative. There was low suspicion for TB pericarditis, as there is no finding on the chest x-ray or fluid analysis report consistent with tuberculosis infection. TB infection would be expected to be lymphocytic predominant in fluid analysis.

Since all microbiological work-up were negative, rheumatological etiology were also investigated. C-reactive marker was elevated but chronic Inflammatory markers such as rheumatoid factor, cyclic citrullinated peptide and antinuclear antibody (ANA) were negative as shown in Table 5. Procalcitonin trended up, and lactic acid gradually improved on adequate fluid resuscitation. The cardiac catheter drainage was removed following repeat echocardiographic finding of stable small epicardial fluid.

**Table 5:** Rheumatology tests performed to rule out other rheumatologist etiology of pleural or pericardiac effusion during week 3 of hospital admission IgA: Immunoglobulin A, IgG: Immunoglobulin G, IgE: Immunoglobulin E, IgM: Immunoglobulin M, dsDNA ab: Double-stranded DNA antibody, SS-A: Anti Sjogren's-syndrome A-related antibody, ANA : Antinuclear antibody, SS-B: Anti-Sjogren's syndrome B-related antibody

Test	Result	Reference	Units
IgG	1349	600-1,640	mg/dl
IgA	193	47-310	mg/dl
IgM	65	50-300	mg/dl
Immunodiffusion IgE	17	<17	ku/L
Rheumatoid factor	<14	<14	lU/mL
Cyclic citrullinated peptide	<16	<16	Units
ANA screen	Negative	Negative	-
Anti-proteinase 3	<1.0	<1.0	AI
SS-A antibody	<1.0 neg	<1.0 neg	AI
SS-B antibody	<1.0 neg	<1.0 neg	AI
Antimyeloperoxidase	<1.0	Negative	-
dsDNA Ab	Negative	Negative	-

Cardiology continued to follow and recommended continued colchicine 0.6 mg twice daily, aspirin 650 mg orally every eight hours for one to two weeks, then tapering down to 325 mg of aspirin orally and pantoprazole for gastrointestinal protective prophylaxis. His chest pain and blood pressures continue to improve overtime. Blood pressure continued to normalize as levophed was gradually titrated down to maintain adequate MAP. Aspirin 81 mg and statin were to be started for coronary artery disease management when the patient was clinically stable. 

**Figure 6:**
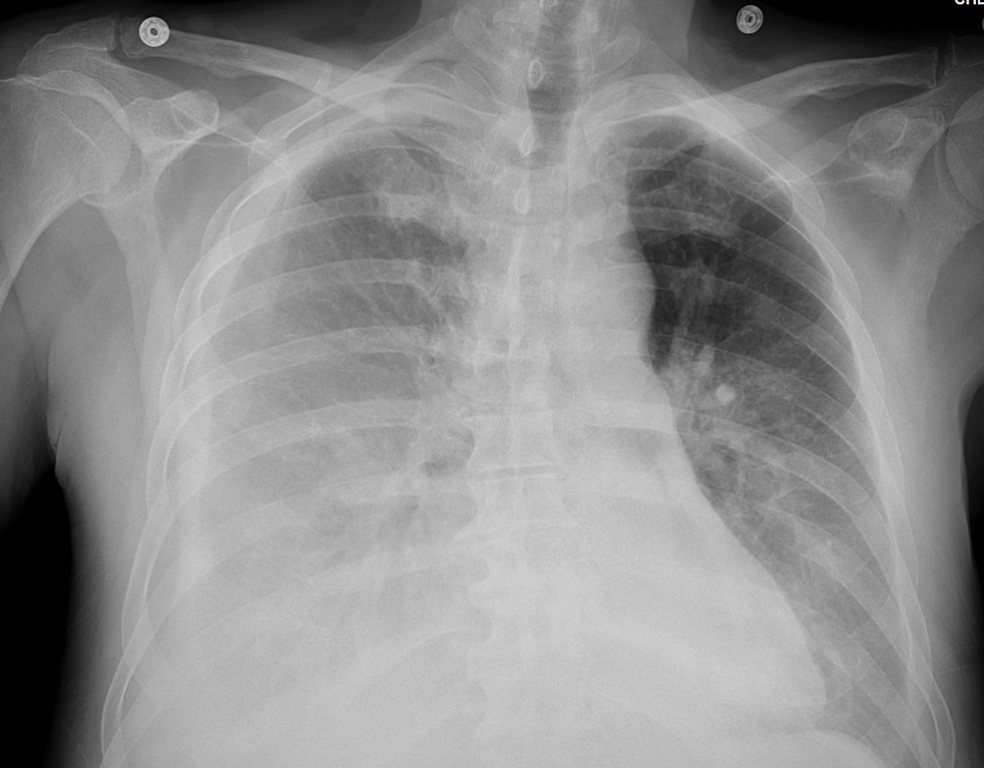
Increasing right-sided pleural effusion (now moderate in size) with associated atelectatic changes with small left-side pleural effusion seen during Week 2 of admission

**Figure 7:**
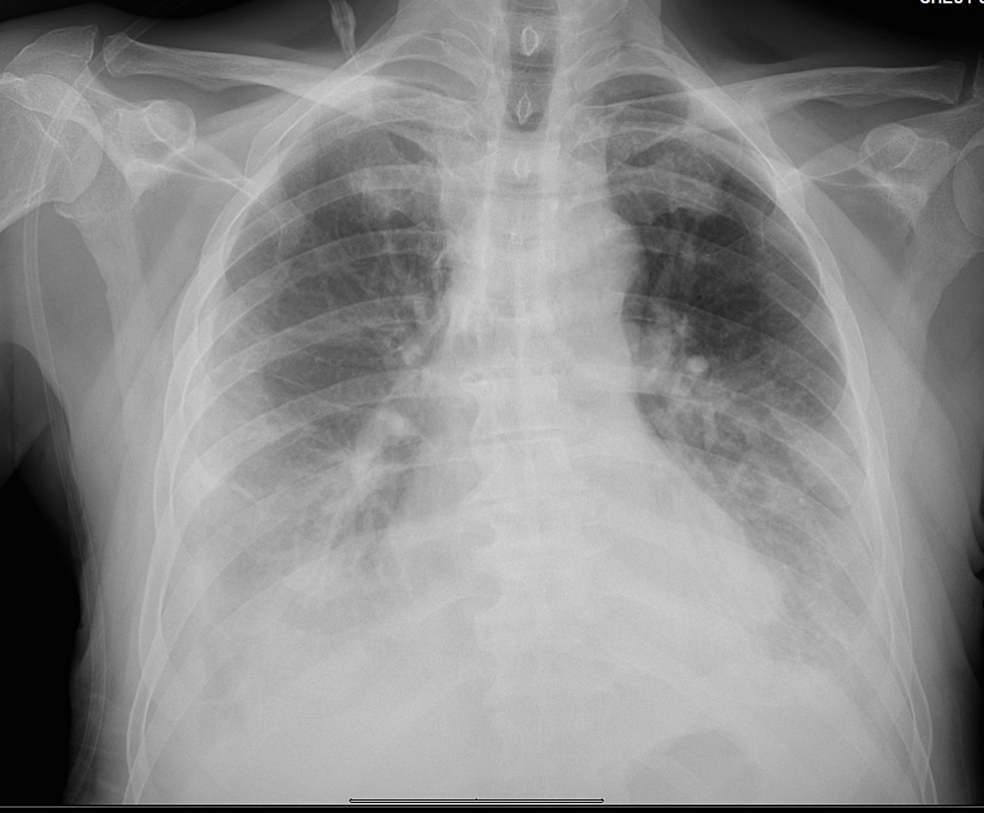
Chest x-ray showing bilateral pleural fluid with left lung fluid increase that developed about a week following the right lung pleural effusion drain

About a week later, he complained of right-sided chest pain, intermittent in nature, sharp and non-radiating, with associated shortness of breath. The pain worsened with movement. physical exam shows decreased breath sound and mild bilateral lung crackles. Chest x-ray showed large right pleural effusion and small left pleural effusion as seen in figure 6. Pulmonology was consulted and the patient underwent thoracentesis. Pleural fluid was sent to the lab for analysis and for cytology as listed in Table 1. Fluid analysis showed exudative specimen. Repeat imaging one week later showed moderate pleural effusion with atelectasis in the left lung. Left pleural fluid increased and was also drained. Cytology of the effusion was negative for malignancy and fluid culture was negative. Repeat chest CT scan with IV contrast showed mild-increasing-moderate pleural effusion re-accumulation as shown in Figure 7. Cardiothoracic surgery was consulted for possible pleural biopsy. Patient was booked for right video-assisted thoracoscopic surgery (VATS), pleural decortication and biopsy the following Monday. 

Given the extended hospital course, acute pericardial effusion, recurrent exudative pleural effusion, with history of RCC three years ago, hematology was consulted to rule out malignancy. CT scan and MRI reviews showed no evidence of recurrence of malignancy. Cancer marker test results were also negative. The patient was transferred to surgery service for VATS. He was taken to operating room and underwent VATS, talc pleurodesis, right pleural biopsy and right chest tube placement. Pulmonology and rheumatology continue to follow up. Right pleural biopsy of a nodule shows necrotic tissue with marked acute inflammation. Patient clinical condition gradually improved and he was discharged. Follow-up visits were arranged with infectious disease, cardiology, pulmonology and primary care doctor.

## Discussion

The pericardium is a fibro-elastic bilayered sac of visceral layer that overlies the epicardium and parietal layer. Both layers of pericardium are separated by a potential space that normally contains 15 to 50 ml of ultrafiltrate of plasma [2]. The pericardium is involved in protection of the heart and it reduces the friction between heart and its surrounding organs. Pericarditis is an inflammatory process involving the pericardial sac, and it accounts for one of the commonest pericardial pathologic processes. "Myopericaditis" is a term used for cases with predominant pericarditis and normal ventricular function. Pericarditis accounts for 0.1% of all hospital admissions and represents 5% of all emergency room visit in patients presenting with chest pain [1,3]. Some data has showed a higher incident rate of acute pericarditis but this may account for only a minority of cases, as many patients with pericarditis are not commonly admitted to the hospital [4,5]. The condition, especially the noninfectious types of it, is frequently seen in men [6]. Most patients who present with acute pericarditis have a benign course of the disease as well as good prognosis. A low percentage of patients present with complications of acute pericarditis. Pericarditis can be classified with consideration of several factors, such as clinical features, course of time and cause. The main type of pericarditis are acute, recurrent, incessant and chronic pericarditis [7,8]. Acute pericarditis accounts for 0.20% of all cardiovascular admissions [7]. The mortality rate for hospitalized patients with acute pericarditis is 1.1 % and increases with age and co-infection severity (pneumonia and septicemia) [7]. About 30% of patients experience recurrence within 18 months following the first episode acute pericarditis [9,10].

Pericarditis is caused by several etiologies such as infections, neoplasms, trauma, autoimmune and metabolic diseases, radiation, drugs and toxins [7,8]. In many cases, idiopathic and viral etiologies are responsible for 80% to 90% of cases in developed countries [1]. Common viral infectious agents include coxsackieviruses A and B, echovirus, adenovirus, Covid-19 virus, and HIV, influenza and herpes viruses [8]. Bacterial causes of pericarditis are infrequent in the developed world and accounts for less than 5% of cases, although tuberculosis is a major cause in developing countries and in HIV patients [11]. Medications, such as procainamine, hydralazine and isoniazid, have been rarely linked as causes of pericarditis [12] . Although our patient had viral symptoms at presentation, all work-up including viral panels via polymerase chain reaction to common viruses were negative. Further workup for other etiologies, including tuberculosis, malignancy, and infections, were negative.

In acute pericarditis, patients present with various symptoms. Clinical diagnosis can be made with two of the following criteria: (i) chest pain, which can be central, severe and pleuritic in nature. Chest pain in pericarditis is characteristically worse with deep breathing and during change in position. Pain is improved by sitting up and leaning forward; (ii) pericardial friction rub; seen in less than 33%; (iii) ECG changes in up to 60% cases, with widespread ST elevation or PR depression during the acute phage; and (iv) mild pericardial effusion seen in 60% of the cases [13,14]. Chest pain may radiate and travel to the ridges of trapezius if the phrenic nerve is inflamed, as it traverses the pericardium [4]. If there is associated myocarditis, as seen in our patient, the pain description will appear to be vague, and heart failure associated symptoms, such as dyspnea, may be present if LV systolic function is majorly affected [15]. Other additional supporting findings depend on the underlying etiology or systemic symptoms such as fever, leukocytosis, elevated inflammatory markers (eg. C-reactive protein, ESR) and evidence of pericardial inflammation by an imaging technique. There are temporal evolutional ECG changes in acute pericarditis that vary from one patient to another. Differential diagnosis such as myocardiac infarction was ruled out as cardiac catheterization did not show evidence of acute coronary syndrome. Chest x-ray is recommended for all patients with suspected acute pericarditis, although usually comes out normal since increased cardio-thoracic ratio occurs if pericardial effusion ratio exceeds 300 ml. In our patient, the initial chest X-ray was contributory and showed an enlarged cardiac silhouette.

The less common identifiable causes, such as non-viral and non-idiopathic, and high-risk features seen in acute pericarditis, have been linked to the associated increased risk of complication such as tamponade, recurrence and constriction [5,15]. Reports of multiple analyses showed that the major risk factor associated with poor prognosis include high fever >100.4°F (>38°C), subacute course, evidence of large pericardial effusion (diastolic echo-free space > 20 mm), cardiac tamponade and unresponsiness to NSAIDs within seven days [10]. Other minor risk factors to consider, especially based on expert opinion and literature reviews, include myopericarditis, oral anticoagulation and immunosuppression [5,8]. For patients presenting with an unusual underlying etiology or with at least one predictor of poor prognosis (major or minor risk factors), they should be triaged and admitted in the hospital for observation, management and investigated for etiology [8,5,16]. Patients without these high-risk features can be managed as out-patients with anti-inflammatory medications and follow up one week later to reevaluate response. Patients with other uncommon etiologies and epidemiological background should be investigated and managed accordingly. Activity restriction to three months (especially competitive sports) until symptom resolution and normalization of CRP and ECG is recommended [8,17,18]. Pericardial complications are grouped under pericardial syndromes. Classical pericardial syndromes are constrictive pericarditis, effusive-constrictive pericarditis, pericardial effusion and cardiac tamponade. 

Below are examples of acute pericarditis complications and complication observed in patients.

Large pleural effusion: In acute idiopathic pericarditis, pleural effusion can co-occur as one of the associated complications but tends to be small and left-sided. Although pleural effusion can be bilateral, it is usually more common in the left pleura and occurs in less than 5% of patients in the right pleura [19,20]. To characterize this type of effusion, about 90% of cases occur in a small portion of the hemithorax and a greater percentage meet light’s exudative criteria. In a retrospective cohort study of patients with pericarditis, 39% had pleural effusion on cardiac MRI [19-21]. Pleural effusions associated with pericarditis are usually small, left-sided or bilateral and exudative [21]. In our case, the patient had a large right pleural effusion with recurrence. It first accumulated on the right side followed with left effusion and subsequent re-accumulation. In another study by Porcel et al., 4% of greater than 3,000 pleural effusions were due to pericardial disease, and it was noted that effusion on both lungs are often in existence in patients with pericarditis but are too small to aspirate and evaluate [8]. The combination of a large pleural effusion with clinical symptoms, the alternating nature and recurrence in our patient is a rare complication, especially since no other etiology is implicated. The pathophysiology of pleural effusion in pericarditis is contiguous inflammation exudation (as pleural and pericardium are in direct contact at the mediastinum) or it could be that the same etiological process affect pleural and pericardium [7,8,19,21]. Further supportive evidence indicating a systemic inflammation role in this process is reported by Thompson et al., who found out that a significant number of patients with various systemic inflammation had pleural and and pericardial involvement [22].

Myo-pericarditis: Pericardial disease associated with myocardial involvement share common etiology, and overlapping forms are totally uncommon. Myopericarditis is referred to as pericarditis with concomitant myocardial involvement. Peri-myocarditis is referred to as predominant myocarditis with pericardial involvement. They present with classical symptoms of pericarditis, and new elevated myocardial injury markers (troponin I or T or CK-MB) without newly developed foci or diffuse impairment in LV function seen in echocardiogram or cardiac MRI [23]. There is rapid elevation in high sensitive troponin in our patient, without significant LV systolic dysfunction symptoms. Many cases are subclinical, although many are preceded by an acute respiratory illness or gastrointestinal symptoms, as seen in our patient [8,24]. Widespread use of troponin and its high sensitivity has contributed to an increased number of reported cases. Although endomyocardial biopsy is confirmatory, with mild LV dysfunction and no additional heart failure symptoms, biopsy is not required [23-25]. Coronary angiography is usually required to rule acute coronary syndrome. Management involves hospitalization and similar treatment as pericarditis. Rest and avoidance of excessive physical activities are recommended in patients with myopericarditis. There has been reported sudden cardiac death in patients after engagement in strenuous activities and this occurs without any prodrome [18]. Physical exercise is contraindicated for six months from the onset of symptoms in patients with myocardial involvement [18]. Myocardial involvement in pericarditis has a good prognosis. Several observation studies show low progression to heart failure or mortality [23,24].

Pericardial effusion/cardiac tamponade: Pericardial effusion has been considered as one of the complications of pericarditis, especially in recurrent pericarditis [20]. Significant pericardial effusion may cause squeezing or constriction of the heart, especially in the right heart chamber due to the thinner wall. This process is called cardiac tamponade, a rare complication of recurrent pericarditis and a serious life threatening condition if not recognized and managed in a timely manner [6]. Cardiac tamponade leads to impaired diastolic filling of the right heart thereby causing venous congestions. It also leads to impaired diastolic filling of the left ventricle which in turn, leads to stroke volume reductions. Cardiac tamponade causes reduced stroke volume, to which the body responds by up-regulating sympathetic effect that increases heart rate and cardiac contractility in order to maintain adequate cardiac output and systemic perfusion. Over time, the blood pressure and cardiac output progressively fail. This leads to multi-organ insult due to shock. As seen in our patient, this obstructive shock leads to decreased perfusion to several end organs, including kidney, liver and brain, thus leading to altered mentation, acute renal failure and shock-related liver disease. Acuteness is more dangerous and leads to rapid onset of symptoms. Compared to a chronic setting, chronic effusion is a gradual accumulation of pericardial fluid of up to 1-2 liters before it can lead to tamponade, giving the parietal pericardium room to stretch to accommodate the abnormal increased volume [4]. The assessment of pericardial effusion and investigation of specific etiological risk type help to have knowledge of complication risk [10,18]. A significant number of patients are asymptomatic and found incidentally via chest x-ray and echocardiogram performed for other reasons. The symptom presentation varies according to speed of accumulation. In an acute setting, even a small amount of increased intra-pericardial pressure can present with symptoms and overt cardiac tamponade within a short time. Slow accumulation takes days to weeks to cause symptoms. Symptom presentation includes shortness of breath on exertion, orthopnea, chest fullness and pain. Patients can also seldom present with nausea, dysphagia, hoarseness and hiccups due to local compressive symptoms. Other non-specific symptoms include fever, cough, weakness and fatigue. When cardiac tamponade develops, patients can present with neck distension, pulsus paradoxus and muffled heart sound on auscultation. CT scan and cardiac MRI are preferred for larger field-view for detection of loculation, pericardial thickening and associated chest abnormality [26]. Treatment of pericardial effusion should be targeted at the etiology and if incited by pericarditis, management should be similar to pericarditis. Patients with cardiac tamponade require emergent pericardiocentesis. In symptomatic patients without evidence of inflammation or failed anti-inflammatory treatment, drainage should be considered. Pericardiocentesis with prolonged pericardial drainage to promote pericardial layer adherence, prevent symptoms and re-accumulation may be considered. NSAID, colchicine and steroids are ineffective in the absence of inflammation. Recurrences are common and may require pericardiectomy or less invasive options like pericardial window following pericardiocentesis [17,26]. The size of effusion is related to etiology and correlates the prognosis. Usually idiopathic etiology with smaller pericardial effusion has better prognosis. But recent studies showed that even mild pericardial effusion is associated with worse prognosis than age and sex-matched controls [27, 28]. Large subacute and chronic effusion, especially unresponsive to therapy, have a high risk of progression to cardiac tamponade especially if they have echocardiographic signs on the right cardiac chamber. This may require pericardial drainage. Six months follow up should be scheduled for moderate idiopathic pericardial effusion. Vasodilators and diuretics are contraindicated in the presence of cardiac tamponade. Constrictive pericarditis is exceedingly rare (<1%) following idiopathic acute pericarditis [10,25]. 

Treatment of pericarditis is usually based on etiology and presence of concomitant diseases considered. Weight-adjusted low-dose colchicine dose is recommended as therapeutic and for recurrence prevention [7]. Colchicine 0.6 mg daily if weight < 70 kg or 0.6 mg 0.6 every 12 hours if weight > 70 kg are used. Addition of tapered high-dose aspirin (750-1,000 mg) or NSAIDs (ibuprofen 600 mg), if aspirin is contraindicated. Although tapering of colchicine is not mandatory, it may be considered to avoid recurrence and symptom persistence [8,15]. Our patient was treated with a tapered dose of colchicine 0.5 mg and aspirin 650 mg every eight hours. Treatment is guided but generally lasts weeks and tapers in one-two weeks in uncomplicated cases. Corticosteroid is a second-line option in patients who have aspirin or NSAIDs contraindications because of risk of chronic evolution of disease recurrence and side effects. When used, low to moderate dose should be recommended, and maintained until symptoms resolution and C-reactive protein normalization and then tapered [27,29,30]. Use of steroids should also be restricted to certain specific indications like systemic inflammatory disease, pregnancy, post-pericardiotomy symptoms and should be avoided if infection (especially TB) is suspected. High-dose steroids should be avoided. In case of recurrence, every effort should be made to avoid dose increase or to reinstate further use. For patients unresponsive to anti-inflammatory therapies or requiring long-term high steroid dose, several other options have been used including azathioprine, IV immunoglobulins (IVIG) and anakira, although no strong evidenced based data back-up [ 9]. Consultation of multidisciplinary experts like rheumatologists, safety measure adoption and patient education of risk involved in immunosuppressive use should be part of treatment plans. As a last resort, pericardiectomy may be considered [27].

## Conclusions

Acute pericarditis is a common pericardial disease with a good prognosis. Few percentile can progress to severe complications. Team effort should be employed in the clinical and radiographic evaluation of patients presenting to health care institutions. A careful triage of high risk factors should guide admission of patients with poorer prognosis. This will help prevent health care morbidity and mortality associated with complicated acute pericarditis.
